# Metagenomic insights into surface sediment microbial community and functional composition along a water-depth gradient in a subtropic deep lake

**DOI:** 10.3389/fmicb.2025.1614055

**Published:** 2025-07-30

**Authors:** Peixuan Zhang, Minglei Ren, Yan Xu, Jianjun Wang

**Affiliations:** ^1^Department of Municipal Engineering, School of Civil Engineering, Southeast University, Nanjing, China; ^2^State Key Laboratory of Lake and Watershed Science for Water Security, Nanjing Institute of Geography and Limnology, Chinese Academy of Sciences, Nanjing, China

**Keywords:** microbial community, functional gene, metagenomic, water depth, deep lake

## Abstract

Deep lakes play a critical role in global elemental cycling and serve as habitats for diverse microbial communities. However, studies on the effects of lake stratification on microbial composition and functional potential in surface sediments remain limited. Here, we investigated microbial community structure and functional composition using metagenomics of 38 surface sediments across a depth gradient of 0–90 m in Lugu Lake, China. Our results showed that Shannon diversity peaked at the thermocline for microbial communities, while a U-shaped pattern for functional genes. Microbial communities and functional genes in the surface sediments showed higher spatial heterogeneity at the shallow layer, whereas those at deeper layers tended toward more homogenized. Although water depth was the most important driver in explaining 29.9 and 26.5% of variance in microbial and functional gene composition, stochastic processes primarily governed the community assemblages, particularly dispersal limitation with the contribution of 43.7%. We further found the surface layer was enriched in genes mainly involved in aerobic metabolism and methanogenesis. In contrast, genes related to reduction reactions, including dissimilatory nitrate and sulfate reduction were more abundant in the thermocline and deep layer, reflecting lower redox potential in a deeper layer. Overall, our results provide evidence for microbial community stratification and functional partitioning in deep lakes.

## Introduction

Deep lakes are critical ecosystems for global biogeochemical cycling, harboring diverse microbial communities that exhibit remarkable adaptability to stratified environmental conditions (Cabello-Yeves et al., 2019). Vertical gradients in temperature, oxygen, and nutrient availability profoundly influence microbial diversity and community assembly processes in the surface sediments across water layers (Wu et al., 2019; Zhao et al., 2019). Surface layers, characterized by dynamic hydrological cycling and higher oxygen levels, support microbes with high dispersal capacity (Lv et al., 2024). In contrast, deeper layers, with reduced oxygen and lower temperatures, impose strong environmental filtering, limiting microbial diversity but fostering specialized microbial assemblages adapted to extreme conditions (Cabello-Yeves et al., 2023). Recognizing microbial community composition and functional potential in surface sediment of deep lakes is essential for revealing their roles in biogeochemical cycles and their responses to environmental changes in the context of accelerating climate change and increasing anthropogenic influence (Falkowski et al., 2008).

Metabolic potential of microorganisms in the sediments also varies across water layers with distinct environmental conditions (Ayala-Muñoz et al., 2022; Haro-Moreno et al., 2018; Imhoff, 2016). Changes in redox status can drive metabolic transitions as depth increases (Bush et al., 2017; Louca et al., 2016; Peura et al., 2015). In the surface layer with abundant oxygen (Chang et al., 2022), microbes in the water columns rely on aerobic respiration and photosynthesis, with genes associated with carbon fixation, glycolysis, citrate cycling and organic nitrogen metabolism being more abundant (Peura et al., 2018; Yin et al., 2019). In contrast, anaerobic respiration and fermentation predominate in oxygen-depleted deep waters, utilizing alternative electron acceptors such as nitrate, sulfate, and carbon dioxide (Lipsewers et al., 2016; Song et al., 2022). Functional genes related to these reductive processes are expected to be more prevalent in the deeper layer, contributing significantly to the metabolic characteristics of the ecosystem (Voss et al., 2013). Although these microbial processes in water columns are well-studied, their variability and interactions with microbial groups in sediment along the depth gradient require further investigation, particularly in subtropical deep lakes.

Here, we collected 38 surface sediments along a depth gradient of 0–90 m in Lugu Lake, a deep plateau freshwater lake. Using metagenomic sequencing, we analyzed the structure and functional profiles of microbial communities and explored the influence of environmental factors including water depth, physical and chemical characteristics. This study aims to address the following three questions: (1) How do surface sediment microbial communities and functional genes vary along depth gradient in the deep lake? (2) What is the relative importance of stochastic and deterministic processes constraining microbial assembly in different layers? (3) Which functional genes in the surface sediment are enriched along the depth of the overlaying water column? (4) How are functional genes coupled with microbial taxa across layers? Our results elucidated the diversity patterns, assembly mechanisms, and metabolic characteristics of microbial communities in Lugu Lake across the depth gradient, providing evidence for stratification of microbial communities and their functional potential in deep lakes.

## Materials and methods

### Field sampling

We collected 38 surface sediments and water samples from 0 to 90 meters in August 2010, in Lugu Lake (27°41′–27°45′N, 100°45′–100°50′E), situated in Yunnan Province, China (Supplementary Figure S1) (Zhao et al., 2023). Lugu Lake is one of the deepest freshwater plateau lakes in the region, with a maximum depth of 93.5 meters and the water surface area of 50.5 km^2^ (Wu et al., 2020). In addition, the lake is situated at an elevation of 2,685 meters, with a catchment area of around 171.4 km^2^ (Zhao et al., 2023). Unlike many lakes in the surrounding area, Lugu Lake remains ice-free throughout the year, due to its warm temperate climate and semi-enclosed nature (Su et al., 2022). During the winter season, the lake exhibits a vertically uniform temperature profile, whereas other seasons feature thermal stratification (Liu et al., 2019). These conditions make Lugu Lake an ideal model for studying microbial community structure and functional enrichment in deep lakes, especially under high-altitude influences.

The detailed procedures for sample collection are described in a previous study (Wang et al., 2012). In brief, three sediment cores with a diameter of 6 cm were retrieved using a gravity core at each site along a water-depth gradient of 0–90 m. The surface sediments were then pooled together. All samples were freeze-dried using a vacuum freeze-dryer and stored at −20°C. Surface water samples were collected from the top 0.5 m of the water column corresponding to the surface sediments collection. Bottom water samples were collected from the sediment–water interface.

### Characterization of environmental factors

We collected and measured a variety of physicochemical parameters of surface, bottom water and surface sediment to investigate the environmental factors influencing microbial communities and functional genes. For surface water, we measure temperature, pH, dissolved oxygen, conductivity, total nitrogen, total phosphorus, concentration of HCO_3_^−^, chlorophyll-a, and silicon content (Wang et al., 2012). For bottom water, temperature, pH, dissolved oxygen, chlorophyll-a and conductivity were measured (Syarif Sukri et al., 2023). For surface sediment, we quantified water depth, total phosphorus, total nitrogen, loss on ignition, porosity, water content, 19 types of metal ions and particle size. The term “water depth” in this study specifically refers to the depth of surface sediments. Detailed methodologies for the measuring and calculating of these abiotic variables have been described in previous studies (Wang et al., 2007). To simplify the complexity of these metal ions, a principal component analysis (PCA) was performed. The first two principal components (PC1 and PC2) were extracted and incorporated as additional environmental parameters for further analysis (Wu et al., 2019; Zhang et al., 2024; Zhao et al., 2019). The details of environmental factors could be found in Supplementary Table S1.

### DNA extraction and metagenome sequencing

Total DNA was extracted from approximately 0.4 g of freeze-dried sediment using the PowerSoil DNeasy Kit (QIAGEN, Germany), with the DNA quality for all samples was evaluated via UV spectrophotometry. Sequencing was performed using a 2 × 150 bp paired-end strategy on the Illumina NovaSeq6000 platform. Raw reads were subjected to quality control and adapter trimming using FastQC and Trimmomatic (Andrews et al., 2010). Reads with average Phred scores below 25 or shorter than 50 bp were discarded. These reads were assembled into contigs with MEGAHIT v1.2.9 using the ‘meta-sensitive’ mode, and the protein-coding genes were predicted by prodigal v2.6.3 from the contigs longer than 1,000 bp (Li et al., 2015). To reduce redundancy, the amino acid sequences of these genes were clustered at the global level using the Linclust algorithm in MMseqs2, with a minimum sequence identity of 0.3 and a fraction of aligned sequences of 0.5, respectively (Hyatt et al., 2010). The relative abundance of the clustered genes was quantified using Salmon v1.0.0, followed by the normalization of total sequencing reads for each gene (Supplementary Table S2). Finally, functional annotation was performed by mapping the clustered genes to the eggNOG 5.0 database with eggnog-mapper v2.1.11, using the DIAMOND v2.1.8 as the search engine (Cantalapiedra et al., 2021; Huerta-Cepas et al., 2019). The genes assigned to the KEGG Orthology (KOs) in the annotation results were aggregated and subjected to the downstream analyses (Andrews et al., 2010). The statistics of assembly and the KO-representing genes across samples were provided in the Supplementary Table S3.

### Community analyses using the rpS3-based approach

Microbial community profiling was performed using a modified pipeline of ribosomal protein rpS3 as described previously (Diamond et al., 2019; Ren et al., 2024). Firstly, the prokaryotic species among the community was represented by the clustering of the conserved marker genes *rpS3* genes from metagenomic assemblies. The marker *rpS3* genes were initially identified from assembled contigs using hmmsearch v3.2.1 (Eddy, 2011) against a custom HMM database, followed by clustering at 99% sequence similarity with USEARCH v11.0.667 (Edgar, 2010) to define species-level operational taxonomic units. Secondly, the relative abundance of the *rpS3*-represented species was estimated through a metagenomic read mapping strategy. The clean reads were aligned to the longest rpS3-containing contigs using Bowtie2 v2.3.5 (Li et al., 2023). The hit reads with ≥99% similarity were counted using the ‘depth’ module of Samtools v1.15.1 (Li et al., 2009). The final abundance of each species in a sample was calculated as the total mapped bases on the representative sequence divided by the representative sequence length and normalized by the total sequencing bases in the sample. Thirdly, taxonomic classification was achieved using the sequence alignment followed by phylogenetic correction. A custom rpS3 reference database was constructed from the RefSeq prokaryotic genome collection (~27,000 genomes, July 2019). Initial taxonomic assignments were generated through BLASTP analysis with the *e*-value threshold 1 × 10^−3^, minimum 50% sequence identity against the reference database. Concurrently, a phylogenetic tree was established through multiple sequence alignment of rpS3 amino acid sequences using MAFFT v7.427 (Katoh et al., 2005), subsequent alignment refinement using trimAl v1.4.1 with automated parameters (Capella-Gutiérrez et al., 2009), and construction of an approximate maximum-likelihood tree using FastTree v2.1.11 (Price et al., 2010). Final taxonomic designations were determined through reconciliation of both complementary approaches, with phylogenetic evidence superseding BLAST-based assignments in cases of discordance or absence of significant database matches.

### Statistical analysis

We conducted a series of statistical analyses to investigate the distribution of microbial communities and functional genes in the surface sediment across water depths. We quantified the alpha diversity of microbial communities and functional genes using Shannon diversity indices (Dixon, 2003) based on the rpS3-based species abundance table and the KEGG Orthology (KO) abundance table at gene level, respectively. To further explore the relationship between Shannon diversity of functional genes and water depth, we applied both linear and quadratic models, selecting the best model based on the lowest Akaike information criterion (AIC) value (Supplementary Table S4) (Spellerberg and Fedor, 2003). For spatial variability (beta diversity), we assessed community composition and functional gene diversity using Bray–Curtis dissimilarity (Gutiérrez-Cánovas et al., 2013). Water depth distances were quantified using Euclidean metrics. To examine the relationship between functional gene beta diversity and depth-related distances, we utilized a Gaussian generalized linear model to analyze distance decay patterns (Supplementary Table S5) (Morlon et al., 2008). The statistical significance of these patterns was evaluated using a Mantel test with 9,999 permutations. Additionally, we performed Non-metric Multidimensional Scaling (NMDS) based on Bray–Curtis dissimilarities to visualize the functional gene composition along the depth gradient.

To explore key drivers of microbial community structure and functional gene distribution, we first applied variable clustering to assess the collinearity among environmental variables, removing redundant variables (Spearman’s *ρ*^2^ > 0.7) to minimize potential confounding effects (Supplementary Figures S2, S3). The relationships between other environmental factors and water depth were presented in supplementary materials (Supplementary Figure S4). In addition, random forest model was employed to determine the optimal number of trees (2,000) using cross-validation and then assessed the importance of each variable (Liaw, 2002). The importance scores were normalized to reflect their relative contribution to the overall model, and we iterated this process, eliminating the least influential variables, until each remaining variable contributed more than 5% to the model (Zhao et al., 2019). Furthermore, we assessed the correlation between microbial community structure, functional gene composition, and environmental variables using Mantel tests with non-redundant variables (Aljohani et al., 2024). In this analysis, Bray–Curtis dissimilarities were used to quantify microbial composition, while Euclidean distances were used for environmental variables.

To further explore the ecological processes shaping microbial communities, we applied a phylogeny-based null model (iCAMP) framework (Ning et al., 2020). This framework utilizes the beta-net correlation index and Raup-Crick index to estimate phylogenetic beta diversity and taxonomic beta diversity (Ning et al., 2020; Stegen et al., 2013). We quantified the relative importance of five ecological processes, including heterogeneous selection, homogeneous selection, dispersal limitation, homogenizing dispersal, and drift. Their differences across water layers were evaluated using the Wilcoxon test (Divine et al., 2013).

To identify microbial functional profiles, we annotated metagenomic reads by comparing them with the Kyoto Encyclopedia of Genes and Genomes (KEGG) database.^1^ To better understand the functional profiles across the three water depth layers, we first identified the functional genes related to carbon, nitrogen, and sulfur cycling (Supplementary Table S3). A generalized linear model (GLM) with a negative binomial distribution was used to estimate differences in gene enrichment between the layers (Robinson et al., 2010). Statistical significance was determined using the Benjamini–Hochberg false discovery rate, with a threshold of *p* < 0.05. GLM modeling was performed using the glmFit function in the edgeR package (Chen et al., 2014). Additionally, to assess the importance of metabolic pathways in community-level metabolic potential and the functional contributions of microbes, we calculated the explained variance of each functional pathway (Supplementary Table S6) and the contribution of microbial genus to these pathways (Supplementary Table S7) (Anantharaman et al., 2016). All *p*-values generated from the Wilcoxon tests were adjusted using the Benjamini–Hochberg false discovery rate method (Chung et al., 2007). All statistical analyses were conducted using R version V4.3.1, with the packages such as vegan V2.6.4, randomForest V4.7.1.1, Hmisc V5.2-3, edgeR V3.30.3, dplyr V1.1.3, ggplot2 V3.4.3 and ggcor V0.9.8.

## Results

### The alpha and beta diversity of taxonomy and functional genes across layers

**Figure 1:**
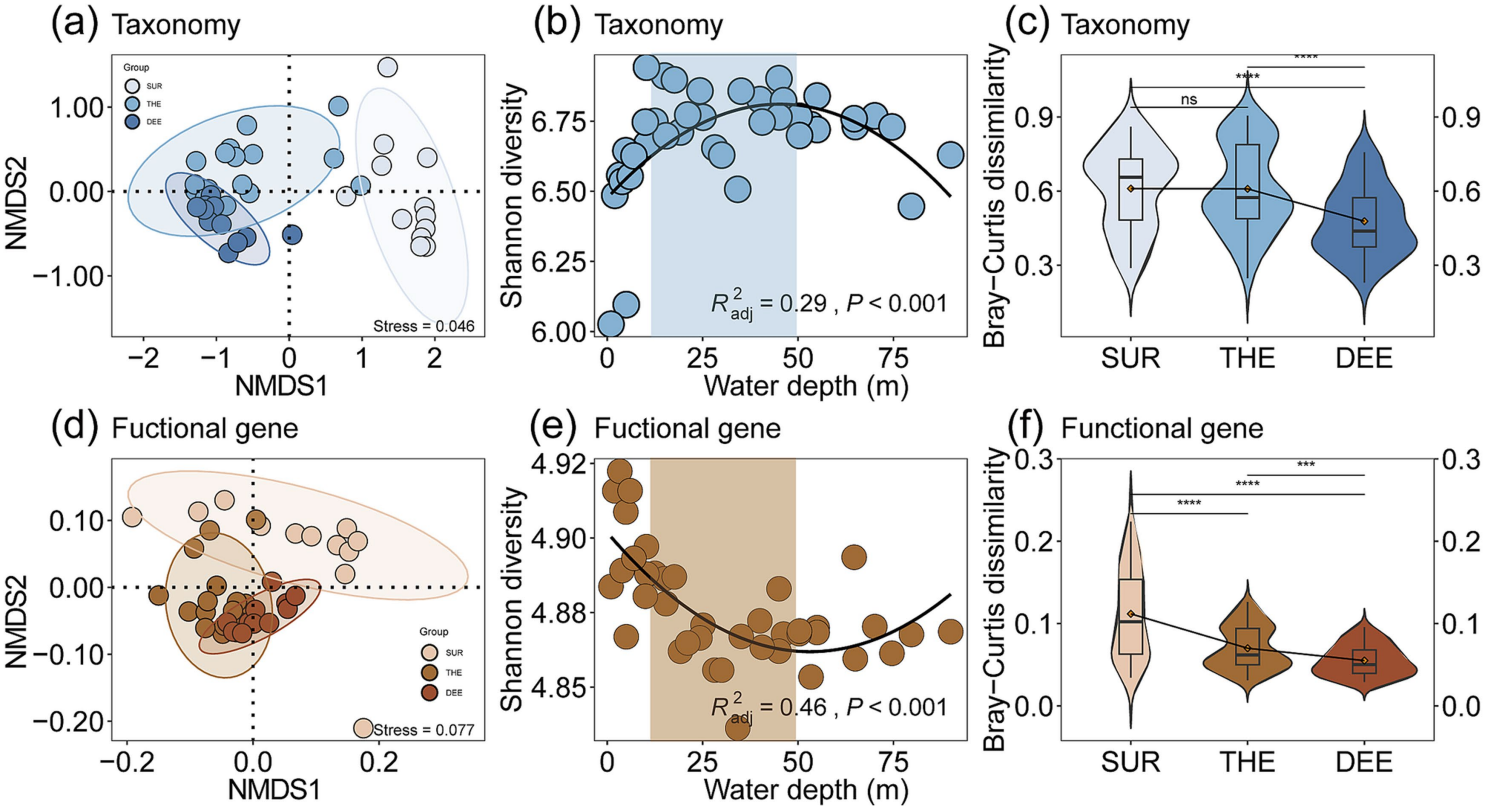
Comparisons of microbial and functional diversity and composition among different water layers. Non-Metric Multidimensional Scaling (NMDS) plots of **(a)** taxonomy and **(d)** functional genes. Each point represents a sample, which was colored by water depth, from surface layer (SUR, 0–10 m) to thermocline (THE, 10–50 m) and then to deep layer (DEE, 50–90 m). The Shannon diversity of **(b)** taxonomy and **(e)** functional genes among three water layers. Differences in microbial beta diversity consisting of **(c)** taxonomic and **(f)** functional genes variation (determined by pair Bray–Curtis distance) among three water layers. Different asterisks in the violin plots denote significant differences in corresponding variables between layer (determined by a two-sided pairwise Wilcoxon test). ^*^p < 0.05, ^**^p < 0.01, and ^***^p < 0.001, and ns: non-significant. In boxplots, the lower and upper hinges of the box correspond to the first and third quartiles (the 25th and 75th percentiles); the upper and lower whiskers extend from the hinge to the largest and smallest values no further than 1.5 times the interquartile range (IQR), respectively; and the central lines represent the median.

**Figure 2:**
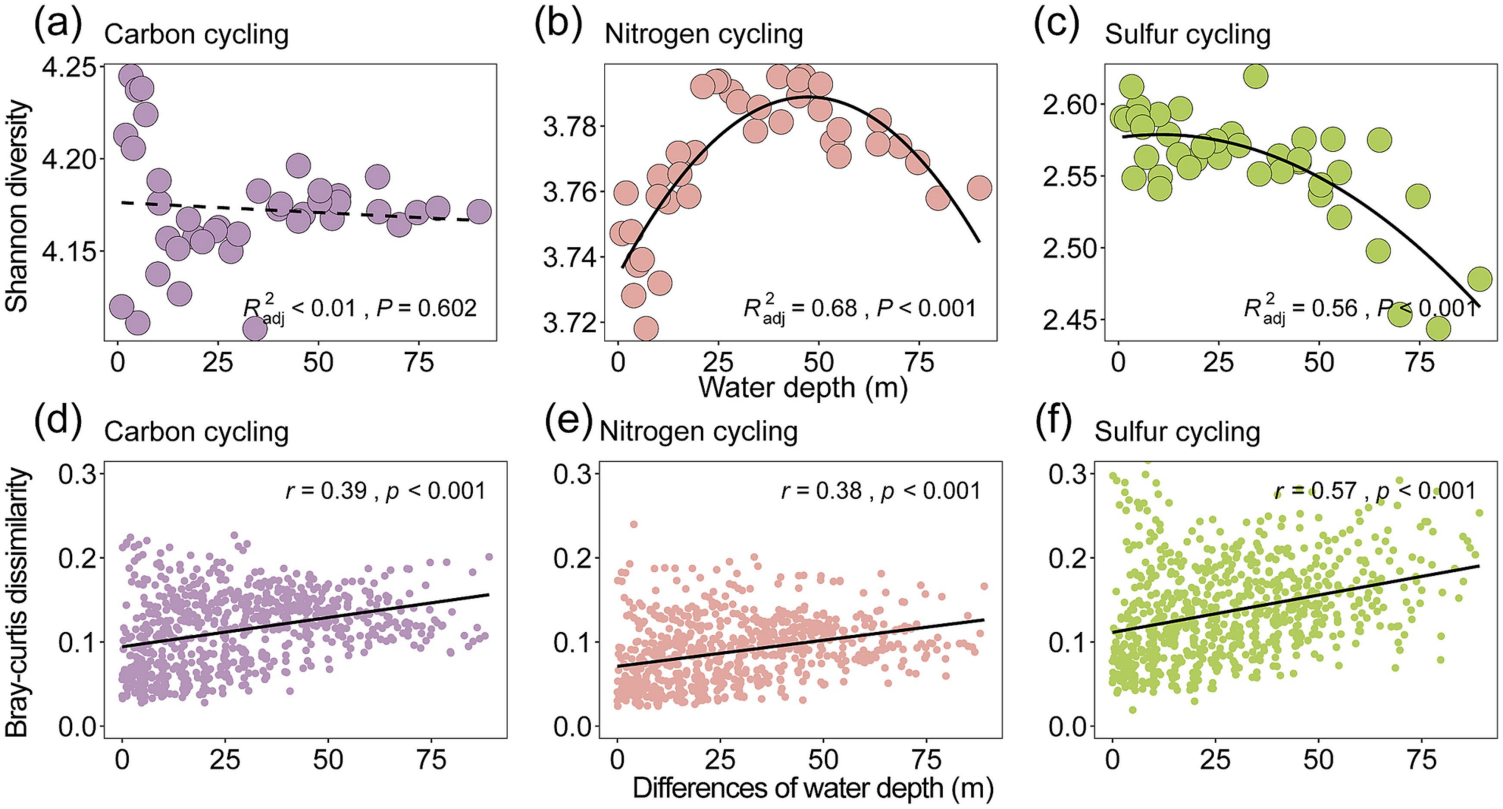
Water-depth diversity patterns and distance-decay relationship for functional genes. We considered the Shannon diversity of the three subgroups of functional genes involved in carbon cycling **(a)**, nitrogen cycling **(b)**, and sulfur cycling **(c)** (Supplementary Table S3). The relationships between functional gene diversity and water depth were evaluated by linear and quadratic models. The better model was selected based on the lower value of the Akaike information criterion. The lower panels **(d–f)** show the relationships between water depth changes and Bray–Curtis dissimilarity of the three subgroups. Linear regressions of relationships based on a linear model are shown with a solid line. Mantel tests were used to examine correlations between differences in functional gene composition and differences in community composition using 9,999 permutations. The Mantel *r*-values are shown, with all *p*-values being less than 0.001. The term “water depth” in this study specifically refers to depth of surface sediments.

The compositions of taxonomy and functional genes in Lugu Lake were associated with the depth of the overlaying water column, with more similar profiles toward deeper layers (Figures 1a–f; Supplementary Figures S5, S6). We further evaluated the Shannon diversity index and β-diversity metrics such as Bray–Curtis dissimilarity of taxa (Figures 1b,c) and functional genes (Figures 1e,f) along depth. Our results showed that microbial Shannon diversity exhibited a hump-shaped pattern peaking at the thermocline (Figure 1b), while functional gene diversity showed a U-shaped pattern along depth (Figure 1e). In addition, our study revealed the three functional subgroups involved in C, N, and S cycling showed distinct patterns (Figures 2a–c and Supplementary Table S4). Specifically, nitrogen cycling genes showed a significant hump-shaped trend along depth peaking around 50 m (*R*^2^_adj_ = 0.68, *p* < 0.001; Figure 2b). In contrast, sulfur cycling genes decreased along with depth, showing a sharp decline beyond 50 m (*R*^2^_adj_ = 0.56, *p* < 0.001; Figure 2c).

**Figure 3:**
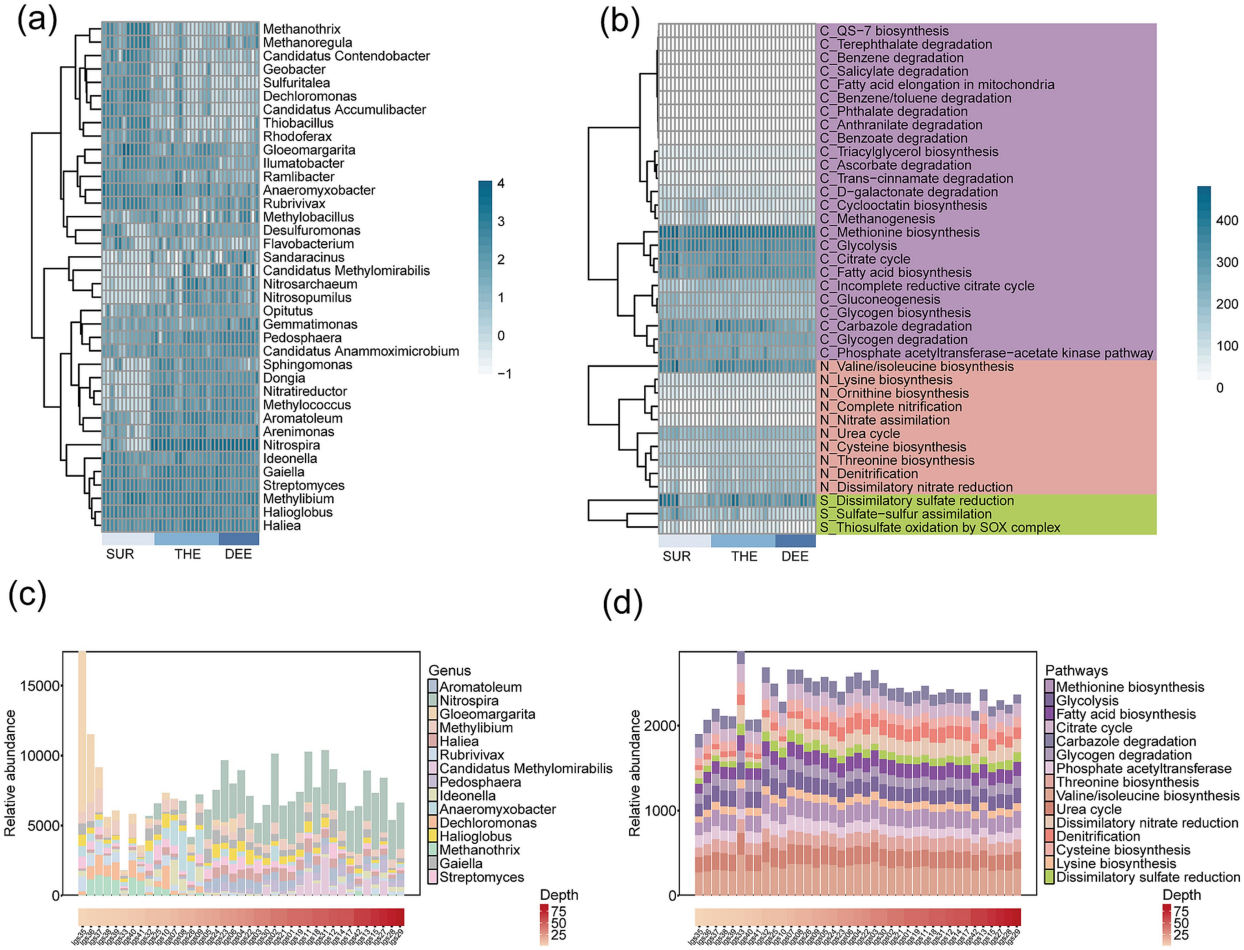
The composition of taxonomic groups and functional traits across different water depths. **(a)** Microbial community and **(b)** functional gene profiles, with samples ordered by water depth. SUR, surface layer; THE, thermocline; DEE, deep layer. Darker colors correspond to higher relative abundances. The relative abundance of **(c)** microbial genus and **(d)** functional pathways in samples from different water depths. Only the top 15 microbial genera with high relative abundances are annotated in the figure.

For beta diversity, the spatial variability of both microbes and functional genes significantly decreased with the depth of the overlaying water column in the deep lake (Figures 1c,f). This indicates distinct changes in the composition of microbial communities and functional genes across different depths and locations. This is supported by the fact that the compositions of microbial communities (Mantel *r* = 0.57, *p* < 0.001) and functional genes (*r* = 0.37, *p* < 0.001) showed a significant distance-decay relationship with water depth changes (Figures 2d–f; Supplementary Figures S7a,b and Supplementary Table S5). The relative abundance of different functional genes remained balanced across samples (Figures 1f, 3b,d; Supplementary Figure S8), compared with microbial communities (Figures 1c, 3c; Supplementary Figure S8).

### Environmental drivers and ecological processes shaping microbial assemblages

**Figure 4:**
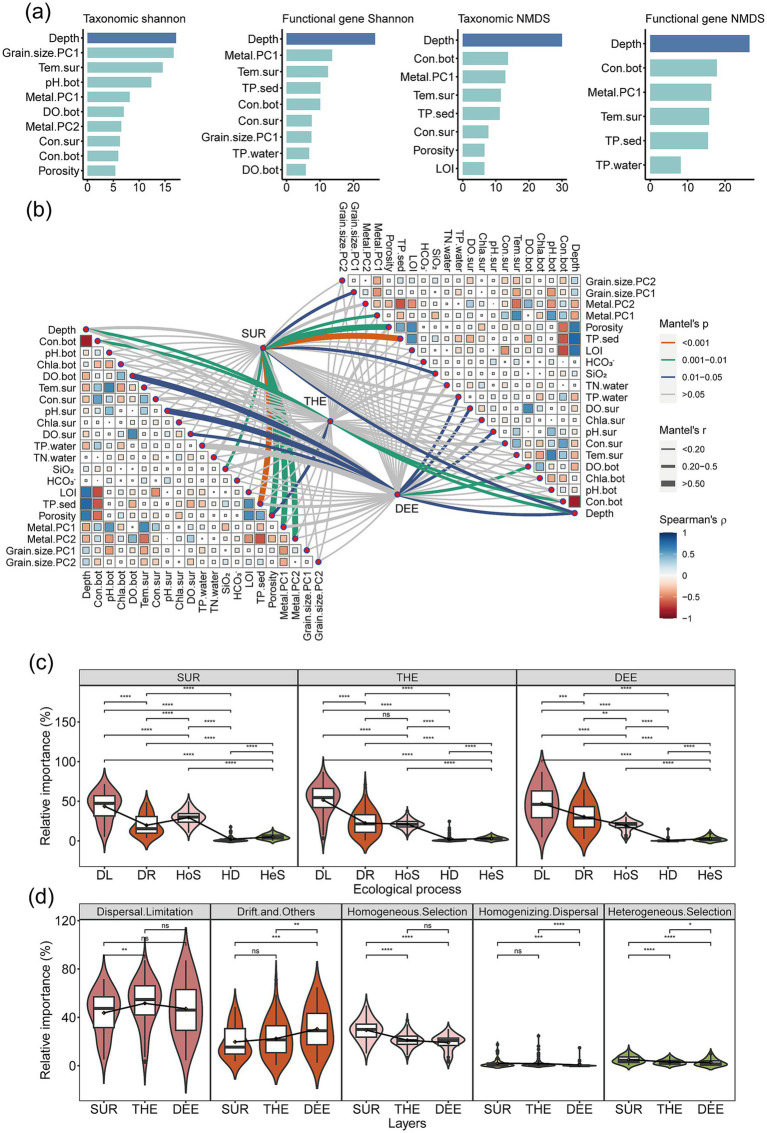
Environmental factors and ecological processes shaping microbial community structure and functional genes. **(a)** Relative contribution of environmental factors to taxonomic and functional diversity. Random forest analysis identified and quantified significant predictors of Shannon diversity and composition. The first axis of NMDS was used to represent composition. We selected the explanatory variables with a relative contribution rate >5%. Details of variable abbreviations are provided in Supplementary Table S1. **(b)** Associations between microbial community structure and functional gene composition (determined by Bray–Curtis distance) with environmental factors (determined by Euclidean distance) using the partial Mantel test. Partial Mantel’s *r* values are indicated by the edge width, while the statistical significance is denoted by the edge color. Pairwise correlations of environmental variables are depicted with a color gradient reflecting Spearman’s correlation coefficient. **(c)** The relative contribution of each ecological process driving microbial community assembly within the layer based on null model analysis (*n* = 231). **(d)** Differences in the relative importance of ecological processes among three water layers (*n* = 231). Different lowercase letters in box plots indicate significant differences for the ecological processes with soil depth (determined by a two-sided Wilcoxon test, *p* < 0.05). SUR, surface layer; THE, thermocline layer; DEE, deep layer. Central line and whiskers in each box represent the median and 1.5 times the interquartile range, respectively. Boxes indicate the interquartile range between 25th and 75th percentiles. Single points are outliers.

We investigated the relative importance of environmental factors in shaping the diversity and composition of microbial communities and functional genes (Figure 4a; Supplementary Figure S9). Water depth had the highest contribution of 17.1% for taxonomic diversity and 26.4% for functional gene diversity. Microbial and functional gene compositions were mainly driven by similar environmental factors, including water depth with 29.9% for microbial composition and 26.5% for functional gene composition, followed by surface temperature and bottom conductivity (Figure 4a; Supplementary Figure S10). Depth was the most influential factor among all environmental factors, distinguishing samples from different depth layers in both taxonomic and functional compositions. Specifically, microbial community structures and functional gene composition in the surface layer were most strongly linked to sediment phosphorus, followed by porosity (Figure 4b). In the thermocline, they were significantly associated with depth, followed by bottom conductivity (Figure 4b). In the deep layer, dissolved oxygen and surface water pH showed the strongest correlations with them (Figure 4b).

Although environmental factors significantly influenced microbial community structure, stochastic ecological processes dominated microbial community assembly in the deep lake as revealed through null model analysis (Figures 4c,d; Supplementary Figure S11). The relative importance of stochastic processes markedly varied with water depth. Specifically, dispersal limitation with an average contribution of 43.7% was the most critical factor in shaping microbial assemblages in the surface layer, followed by homogeneous selection with 29.5% (Figure 4c). In the thermocline and deep layers, dispersal limitation remained the primary mechanism influencing microbial community assembly with 51.6 and 47.1%, followed by drift with 22.4 and 30.4%, respectively (Figure 4c).

### Enrichment of layer-specific C, N, and S cycling gene

**Figure 5:**
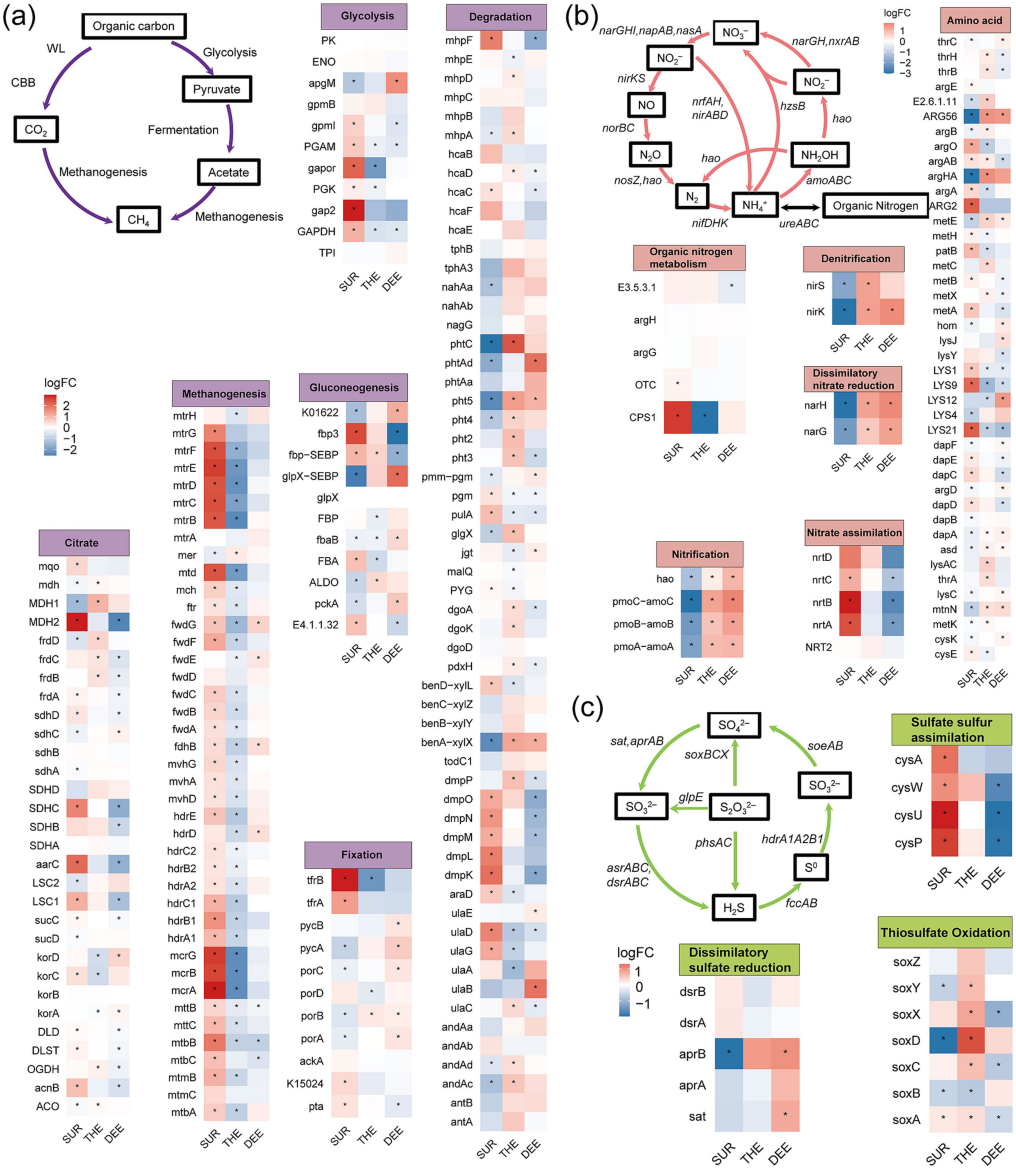
Differences in the abundance of functional genes involved in C, N, and S cycling across three layers. The heatmap shows the enrichment of functional genes involved in **(a)** carbon cycling, **(b)** nitrogen cycling, and **(c)** sulfur cycling among three water layers. Statistical significance of the changes in gene abundance was assessed by a generalized linear model with a negative binomial distribution using edgeR package. The *p*-values were obtained from two-sided likelihood ratio tests (LRTs) and adjusted for multiple comparisons via the Benjamini–Hochberg procedure. Genes with significant changes in abundance (*p* < 0.05) are indicated with an asterisk. LogFC, log2-fold change. The full names of the genes in this figure are listed in Supplementary Table S3. SUR, surface layer; THE, thermocline layer; DEE, deep layer.

Our results revealed significant enrichment in the composition of functional genes involved in carbon, nitrogen, and sulfur cycles across different layers (Figures 5a–c; Supplementary Figure S12 and Supplementary Table S3). Genes enriched in the surface layer were predominantly associated with rapid energy acquisition and organic matter degradation. We found that the genes involved in the tricarboxylic acid (TCA) cycle, glycolysis, and methanogenesis were enriched in the surface layer (Figure 5a). The abundance of methanogenesis-related genes (*mcrA*, *mtrB*) was higher in the surface layer (Figure 5a). Additionally, genes involved in nitrate assimilation (*nrtABC*) and organic nitrogen metabolism (*CPS1*) were more abundant in the surface layer, suggesting that surface microbes preferentially utilize inorganic nitrogen and organic nitrogen sources (Figure 5b). Genes related to thiosulfate assimilation were also enriched in the surface layer, suggesting that surface microbes assimilate sulfur to support higher primary productivity and organic sulfur demand (Figure 5c). Genes involved in anaerobic metabolic pathways or reduction reactions were more abundant in the deeper layers. The thermocline and deep layer were enriched in genes associated with nitrification (*amoA*, *hao*), denitrification (*nirS*, *nirK*), and dissimilatory nitrate reduction (*nafH*, *narG*) (Figure 5b). Genes related to sulfide oxidation (*soxACDXY*) were enriched in the thermocline. Additionally, phthalate degradation genes were more abundant in the thermocline. The deep layer was enriched in genes associated with dissimilatory sulfate reduction (*aprB*, *sat*).

### Contribution of microbial taxa to C, N, and S cycling genes across the layer

**Figure 6:**
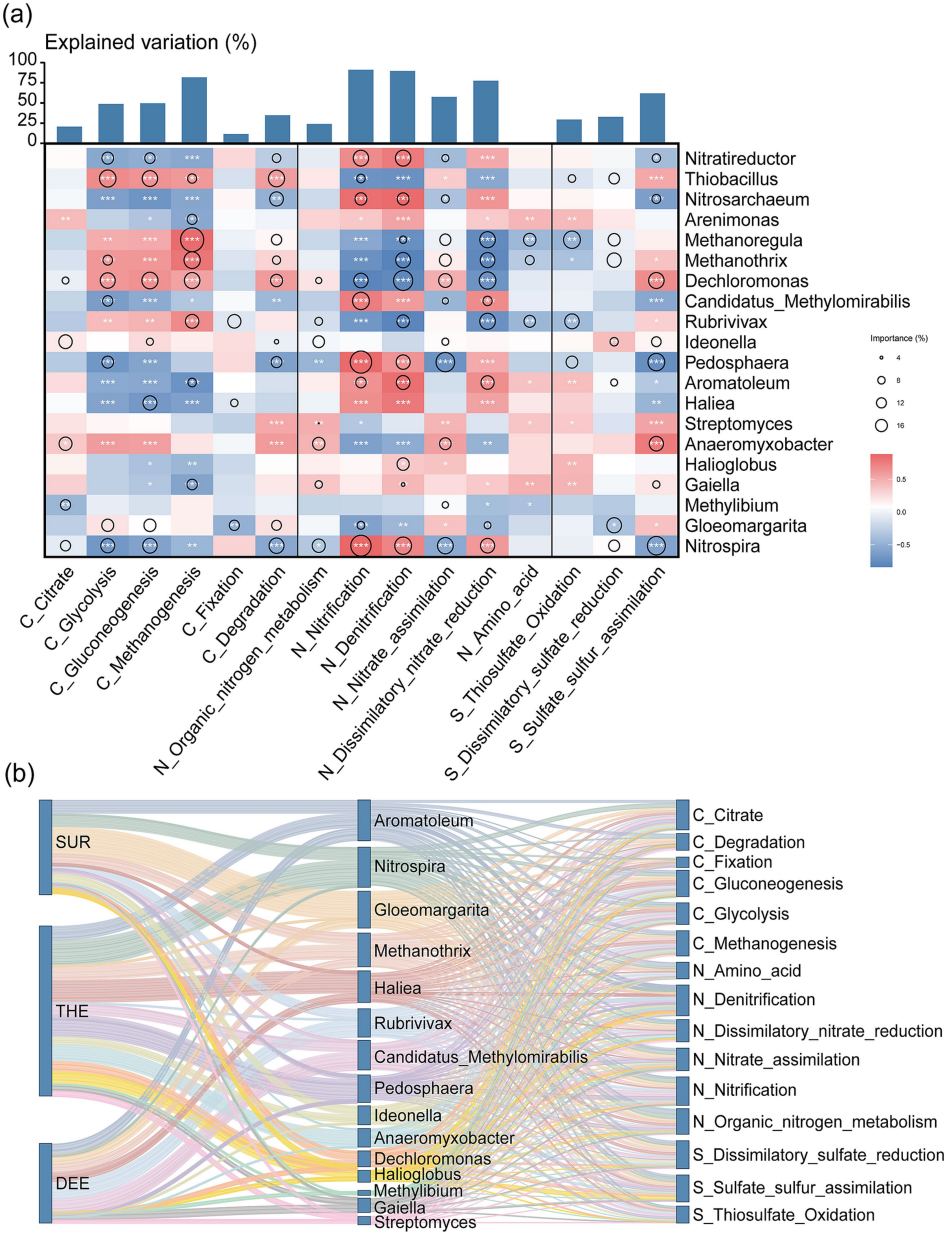
Contribution of microbial communities to biogeochemical processes across different water layers. **(a)** The contribution of microbial genus to each metabolic pathway through random forest analysis is represented by circles of different sizes. The color gradient reflects the strength of the Spearman correlation coefficient, where dark blue indicates a strong positive correlation, and dark red represents a strong negative correlation. Statistical significance is denoted by asterisks: ^***^p < 0.001, ^**^p < 0.01, and ^*^p < 0.05. **(b)** The Sankey diagram illustrates the difference in the contributions of microbial groups to specific biogeochemical processes across three water layers, with the taxonomic classification of microbial groups and their associated category of functional pathways. The three columns represent, from left to right, water layers, taxonomic groups, and metabolic pathways, respectively. SUR, surface layer; THE, thermocline layer; DEE, deep layer.

To evaluate the relative importance of metabolic pathways in the deep lake and the contribution of microbial taxa to metabolism, we quantified the explained variance of each metabolic pathway and assessed the relative contributions of various microbial phyla (Figure 6a and Supplementary Tables S6, S7). Pathways with higher explained variance were considered more critical to community-level metabolism. Among the metabolic pathways, nitrification emerged as the most critical process with explained variation of 91.07%, followed by denitrification with 89.56%, methanogenesis with 81.72%, and dissimilatory nitrate reduction with 77.45% (Figure 6a and Supplementary Table S6). This demonstrates the important roles of carbon, nitrogen, and sulfur cycles in regulating microbial energy metabolism in the lake. In the surface layer, metabolic activity was predominantly driven by *Nitrospira*, *Gloeomargarita* and *Methanothrix*, (Figure 6b and Supplementary Table S7). The thermocline exhibited more diverse microbial communities, with significant contributions from *Nitrospira*, *Haliea*, *Methanothrix*, *Pedosphaera* and *Anaeromyxobacter* (Figure 6b and Supplementary Table S7). In the deep layer, *Aromatoleum*, *Rubrivivax* and *Candidatus Methylomirabilis* were the primary contributors to functional cycling (Figure 6b and Supplementary Table S7).

## Discussion

Using metagenome sequencing technology, we identified four main findings: (i) The diversity of microbial communities and functional genes exhibited distinct depth patterns, with microbial Shannon diversity exhibiting a hump-shaped pattern and functional gene diversity showing a U-shaped pattern; (ii) Despite water depth significantly driving microbial and functional gene composition, stochastic ecological processes such as dispersal limitation dominated community assembly in the deep lake; (iii) Functional gene enrichment analysis revealed that the surface layer was primarily linked to aerobic respiration and methanogenesis, while the thermocline and deep layers were enriched in genes associated with anaerobic metabolism; (iv) The metabolism at the surface layer was contributed by taxa that thrive on light and oxygen for rapid energy acquisition and decomposition, while deeper layers shifted to anaerobic metabolism.

### Taxonomic and functional diversity across layers

The thermocline effectively separated the mixed or surface waters from the colder bottom layers (Chang et al., 2022). Oxygen levels fluctuate in the thermocline region of Lugu Lake, and there are steep gradients of temperature and redox potential (Ren and Wang, 2022; Tran et al., 2021). Frequent sediment resuspension events potentially altering redox gradients in the surface sediments (Dadi et al., 2017; Nevers et al., 2020). This unique environment creates diverse microhabitats, providing opportunities for different microbial communities to thrive (Xing et al., 2019). In the deep layer, the challenging conditions of sediments such as low temperatures, hypoxia, and nutrient scarcity restricted microbial growth, potentially leading to a decrease in microbial Shannon diversity (Cabello-Yeves et al., 2019). In the thermocline region, steep oxygen gradients made nitrogen cycling genes diverse and active (Chang et al., 2022). The dynamic environment of this layer facilitated the coexistence and functional differentiation of nitrogen-cycling microbes, leading to peak functional gene diversity (Martens-Habbena et al., 2009). In the deep and oxygen-deprived layer, sulfur cycling shifted toward sulfate reduction, a process typically dominated by specific taxa like sulfate-reducing bacteria (Chang et al., 2022; Zhang et al., 2024). The harsh conditions imposed strong selective pressure, allowing well-adapted microbes carrying specific functional genes (e.g., *dsrAB*, *aprB*, *sat*) to persist, thus limiting sulfur cycling functional gene diversity (Müller et al., 2015; Wasmund et al., 2017).

Microbial communities of sediments in the surface layer exhibited higher spatial heterogeneity, while those in the deeper layers tended to be more homogenized. This trend aligned with previous research showing that the total beta diversity of microbial communities in the sediments showed significant decreasing trends toward deeper water in deep lake sediments (Wu et al., 2020). The homogenization in deep layers can be attributed to the unique physicochemical characteristics of deep lakes, such as depth-specific environmental gradients and limited nutrient availability, which restricts species exchange and diffusion between microbial communities in the water columns at different depths (Lear et al., 2014). Microbial communities in the surface layers are more influenced by nutrient exchange, fostering a more varied ecological environment (Wang et al., 2022).

### Environmental drivers and ecological processes shaping microbial assemblages

The responses of microbial communities and functional genes to environmental factors were strongly coupled, contrasting with previous studies in soil and ocean where environmental factors strongly influenced functional groups but had a weaker effect on community composition (Louca et al., 2016). Depth and temperature were identified as robust predictors of microbial communities and functional genes of the sediments in lake ecosystems (Zhang et al., 2024; Zhao et al., 2019). As a multifaceted proxy, depth encapsulated variations in temperature, dissolved oxygen, light availability, and nutrient levels, influencing microbes directly or indirectly (Kitazawa et al., 2018; Nevalainen, 2012; Rojas-Jimenez et al., 2021). These environmental gradients modulated resource availability, energy-related processes, and stressor prevalence, exerting selective pressure on microbial communities and functional composition (Gurung et al., 2001; Wu et al., 2020).

Despite the relatively open nature of the surface environment, spatial resource heterogeneity imposed significant limitations on microbial dispersal (Martiny et al., 2006). Homogeneous selection was likely linked to the strong selective pressures exerted by key environmental factors such as light availability and dissolved oxygen concentration (Gotelli, 2000; Liu et al., 2020; Zhao et al., 2019). The thermocline, characterized by the steepest temperature changes within the water column, may constrain microbial migration and dispersal by altering water density and viscosity (Morrison et al., 2017). This pronounced these temperature changes created a strong physical stratification, impeding material exchange between upper and lower water layers and restricting microbial migration across different depths (Peura et al., 2015; Zhang et al., 2024). The deep layer was characterized by stable but harsh physicochemical conditions, limiting the ability of different microbial species to colonize and establish diverse communities (Wang et al., 2013). Furthermore, the importance of drift increased significantly with depth (Figure 4d), likely due to the scarcity of resources and smaller community sizes in deeper layers (Ofiţeru et al., 2010). These factors amplified the impact of random population fluctuations on community structure. We also found homogenizing selection decreased with depth (Figure 4d), reflecting the diminishing influence of environmental factors such as light and oxygen (Wang et al., 2013; Zhao et al., 2021). The effects of environmental filtering weakened, and stochastic processes became more dominant in shaping community structure as depth increases.

### Functional enrichment of C, N, and S cycling genes across layers

The enrichment of genes associated with energy acquisition and organic matter degradation in the surface layer suggests that microbial communities in this zone are adapted to the oxygen-rich, light-rich conditions where rapid energy cycling is critical for supporting primary productivity (Cunliffe et al., 2008; Peura et al., 2018). Moreover, the high abundance of methanogenesis-related genes in the surface layer may be attributed to the presence of localized anaerobic microenvironments, such as particle deposition zones, which facilitate methane production processes (Bridgham et al., 2013). The preferential utilization of inorganic and organic nitrogen sources by surface microbes highlights the competitive pressures in this layer, where primary producers like phytoplankton dominate and microbial communities must efficiently assimilate available nitrogen to sustain their growth (Chrost et al., 2009). Similarly, the enrichment of sulfur-related genes suggests that surface microbes also play a role in supporting primary productivity by assimilating sulfur, an essential nutrient for microbial growth and metabolic processes.

The enrichment of genes involved in anaerobic metabolic processes in the deeper layers suggests a shift toward energy pathways that do not rely on oxygen, as microbes adapt to low-oxygen conditions in these layers. The presence of genes related to nitrification, denitrification, and nitrate reduction in the thermocline and deep layers indicates that microbes in these layers rely on anaerobic nitrogen transformations, such as nitrate and nitrite reduction, to adapt to the limited oxygen availability in deeper waters (Robert Hamersley et al., 2009). The enrichment of genes related to sulfide oxidation in the thermocline was probably due to the elevated sulfide concentrations at the redox interface (Chen et al., 2024). Microbes in this layer may oxidize sulfide to generate intermediate sulfur compounds, to adapt to the oxidative conditions of this transitional environment (Zhang et al., 2021). Phthalate degradation genes were more abundant in the thermocline. Phthalates and their derivatives, common pollutants, may originate from sediment release or external inputs (Tuan Tran et al., 2022). The sharp temperature gradient and stable chemical stratification of the thermocline created a unique ecological niche supporting the degradation of complex organic compounds (Kurt, 2019). Microbes in this layer may leverage adaptive metabolic capabilities to selectively degrade structurally complex organic carbon, contributing significantly to the carbon cycle in this stratified environment. The enrichment of genes associated with dissimilatory sulfate reduction in deep layer reflected microbial reliance on sulfate as a terminal electron acceptor for anaerobic respiration (Zhu et al., 2018). This metabolic trait corresponded to the anoxic conditions of the deep layer, where sulfate reduction serves as a critical energy-yielding process (Watanabe et al., 2013).

### Layer-specific contribution of microbial taxa to functional genes

The surface layer, characterized by high organic carbon and nutrient availability, supports robust microbial activity (Ertefai et al., 2008). *Gloeomargarita*, an oxygenic photoautotroph, contributed to both photosynthesis and organic carbon degradation (Bacchetta et al., 2022; Moreira et al., 2017). Its distribution aligns with previous observations in soil (Nelson et al., 2016) and marine systems (Zehr and Capone, 2020), suggesting that *Gloeomargarita* distribution is strongly influenced by factors such as light intensity, temperature, and nutrient availability. Its predominance highlights the importance of oxygenic metabolism and primary production in surface waters, where light and oxygen are readily available.

*Nitrospira*, as a key nitrifying genus, indicates high nitrogen turnover in the thermocline, suggesting active nitrification processes (Ren and Wang, 2022; Winter et al., 2009). The thermocline with its steep temperature gradient and variable oxygen levels, supports both aerobic and anaerobic metabolic processes (Gorham and Boyce, 1989). *Haliea* commonly exhibited photoheterotrophic traits, indicating the persistence of light-driven energy capture in the thermocline (Yamamoto et al., 2020; Yang et al., 2020). These metabolic activities likely provide additional energy for microbial processes in the thermocline. The deep layer, characterized by low oxygen and nutrient availability, exhibited significant anaerobic and heterotrophic metabolic activities. *Aromatoleum* remained dominant in the deep layer, utilizing its ability to metabolize organic substrates under anaerobic conditions (Becker et al., 2022). Its metabolic strategies likely reflect adaptations to limited energy resources, emphasizing the importance of efficient resource utilization in deep lake environments (Vagts et al., 2018).

## Conclusion

Our study provides metagenomic analyses of microbial community structure and functional potential along a water depth gradient in Lugu Lake, a deep lake in subtropic zone. We revealed significant shifts in microbial diversity and functional gene composition across the lake sediment in response to the depth of the overlaying water column and its redox state, revealing tight metabolic coupling between sediment and water column ecosystems. Microbial communities in the shallower layers exhibited higher spatial heterogeneity, while those in the deep layers were more homogenized. The thermocline, with steep gradients of temperature and redox potential, created diverse microhabitats that support different microbial lineages to thrive. We also observed that microbial communities involved in alternative electron accepting processes were more diverse in the thermocline, likely due to the lower redox potential and complex nutrient strategies in this layer.

Functional genes involved in carbon, nitrogen, and sulfur cycling showed layer-specific enrichment. Surface waters were dominated by taxa that exploit abundant light and oxygen, favoring rapid energy acquisition and organic matter decomposition. In contrast, the thermocline and deep layers shifted toward anaerobic and specialized metabolic pathways, reflecting adaptations to oxygen-limited and nutrient-poor conditions. The diverse metabolic strategies observed across depth gradients underscore the critical role of microbial communities in regulating biogeochemical cycles in deep lakes. These findings emphasize that the thermocline significantly affects the shaping of microbial community and functional gene distributions. Future research could focus on multi-season sampling to capture annual variability in microbial dynamics and ecosystem processes.

## Data Availability

The metagenomic data presented in this study have been deposited in the National Omics Data Encyclopedia (NODE, https://www.biosino.org/node/index) under the accession number OEX011297 (experiment ID).

## Footnotes

^1^ http://www.kegg.jp
