# Supplementary material for: Metagenomic insights into surface sediment microbial community and functional composition along a water-depth gradient in a subtropic deep lake
Source: Front Microbiol. 2025 Jul 30;16:1614055. doi: 10.3389/fmicb.2025.1614055 (PMC12343629; doi:10.3389/fmicb.2025.1614055)
Supplement: Supplementary file 1 [file Supplementary_file_1.docx]

**Supplementary materials**

**Metagenomic insights into stratification of microbial community and functional composition in a subtropic deep lake**

Peixuan Zhang^1,2^, Minglei Ren^2,*^ , Yan Xu^1,*^, Jianjun Wang^2^

^1^ Department of Municipal Engineering, School of Civil Engineering, Southeast University, Nanjing 210096, China

^2^ State Key Laboratory of Lake and Watershed Science for Water Security, Nanjing Institute of Geography and Limnology, Chinese Academy of Sciences, Nanjing 211135, China.

^*^E-mail: [mlren@niglas.ac.cn](mailto:mlren@niglas.ac.cn); [xuxucalmm@seu.edu.cn](mailto:xuxucalmm@seu.edu.cn)

**Running title:** Metagenomic insights into functional genes in deep lake

**Keywords:**

Microbial community, Functional gene, Metagenomic, Water depth, Deep Lake

**Supplementary Figures**


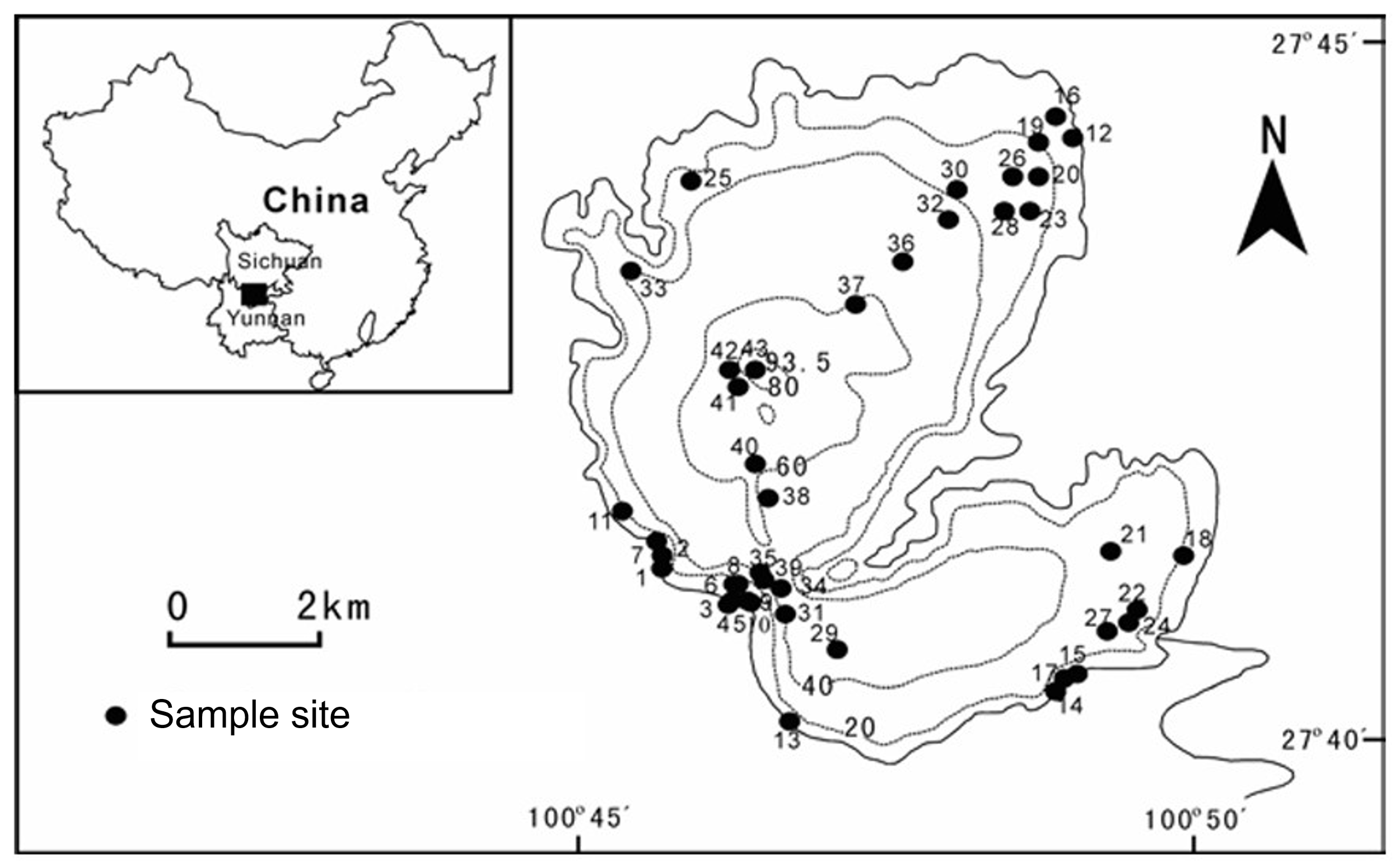


**Figure S1**. **Geographical distribution of the study area.** Map of China indicating the position of Sichuan and Yunnan provinces and the geographical location of Lugu Lake. Bathymetric map detail showing the distribution of surface sediment sampling sites along a depth gradient in Lugu Lake (modified from Wang et al., 2012).


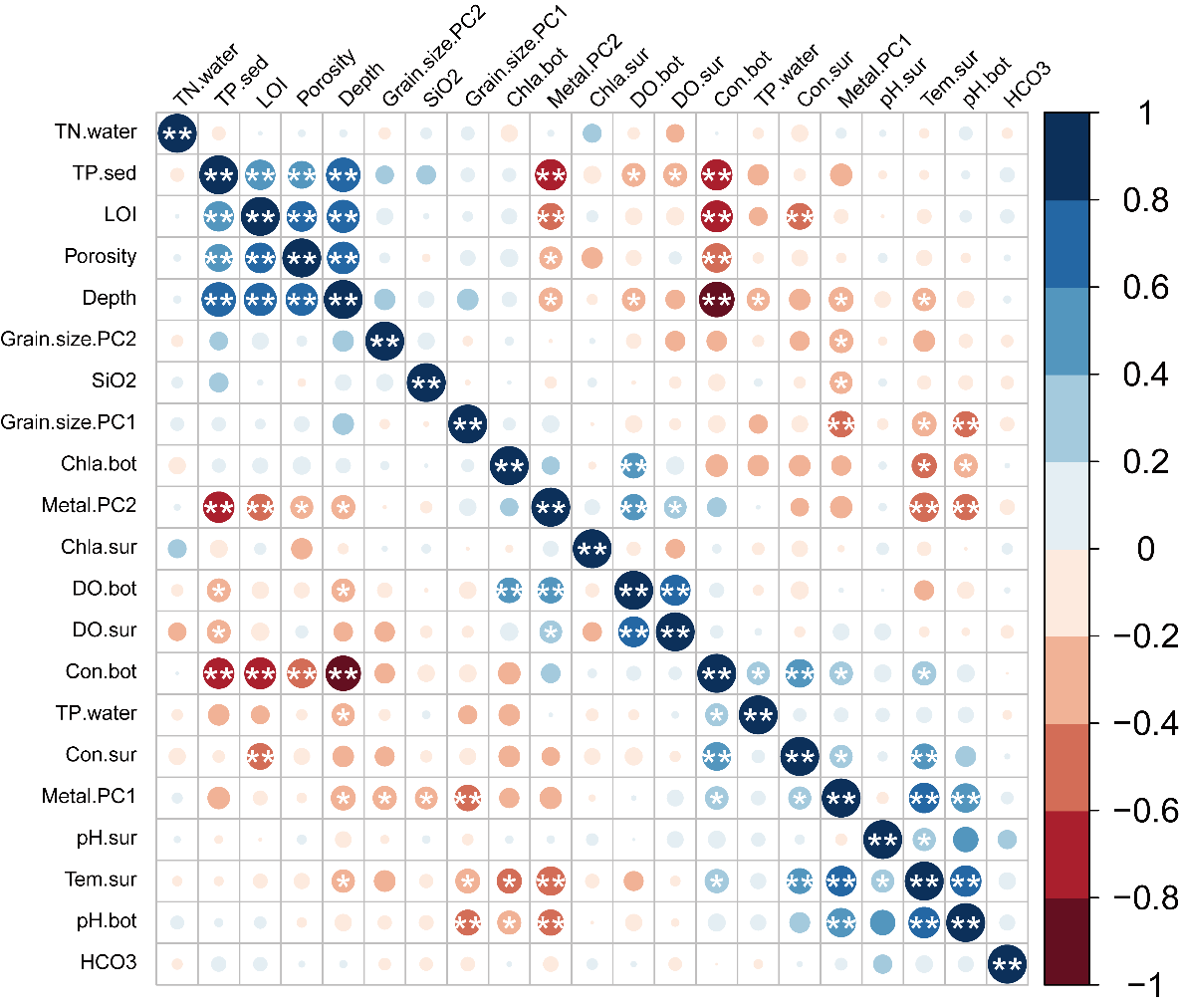


**Figure S2**. **Pearson's correlation heatmap analysis of environmental factors.** The blue and red colours show positive and negative relationship between the two variables, respectively. The colour is darker, the relationships are stronger. The colour is proportional to the Pearson's correlation coefficients (dark blue, *r* = 1, dark red, *r* = -1). (** indicates *p* < 0.01; * indicates *p* < 0.05). Table S3 provided details of the abbreviations of the explanatory variables.


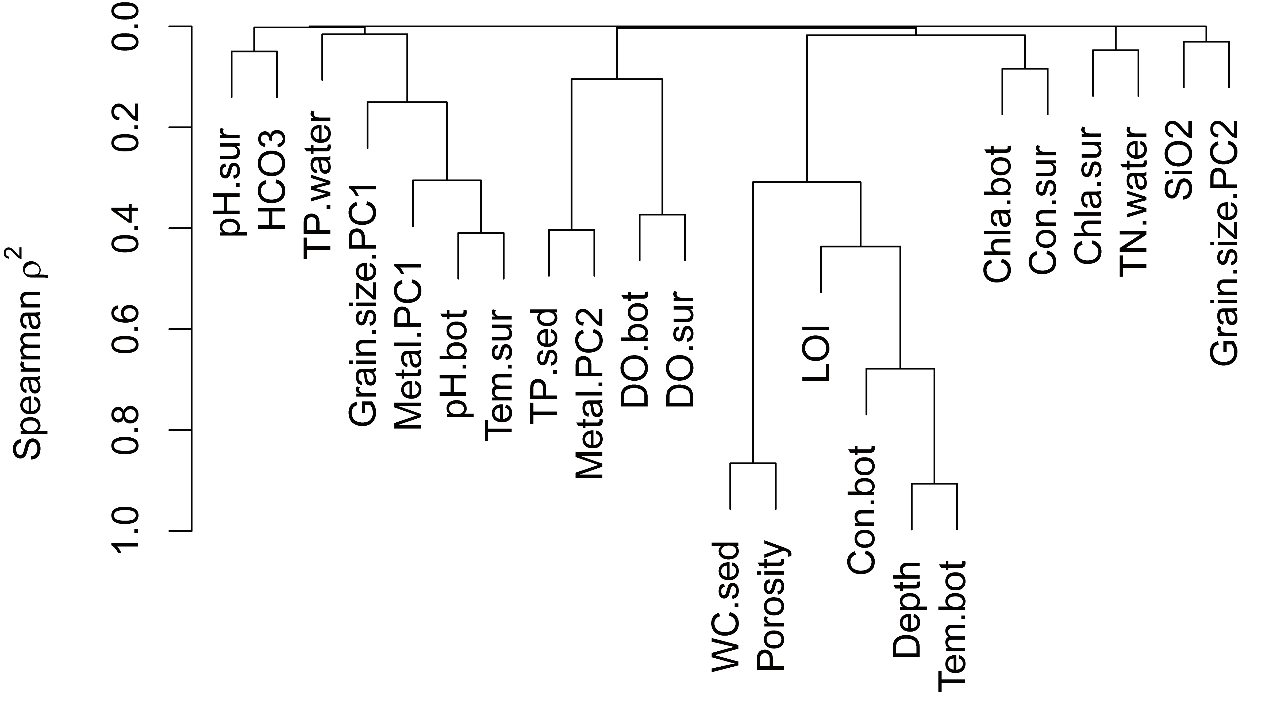


**Figure S3**. **Clustering analysis of environmental variables to reduce redundancy.** The clustering analysis was performed using the varclus procedure in the Hmisc R package to identify and remove redundant variables (Spearman’s *ρ²* > 0.7). Variables with a high correlation (Spearman’s *ρ2* > 0.7) were removed to avoid redundancy. Details of environmental variables could be found in Table S3.


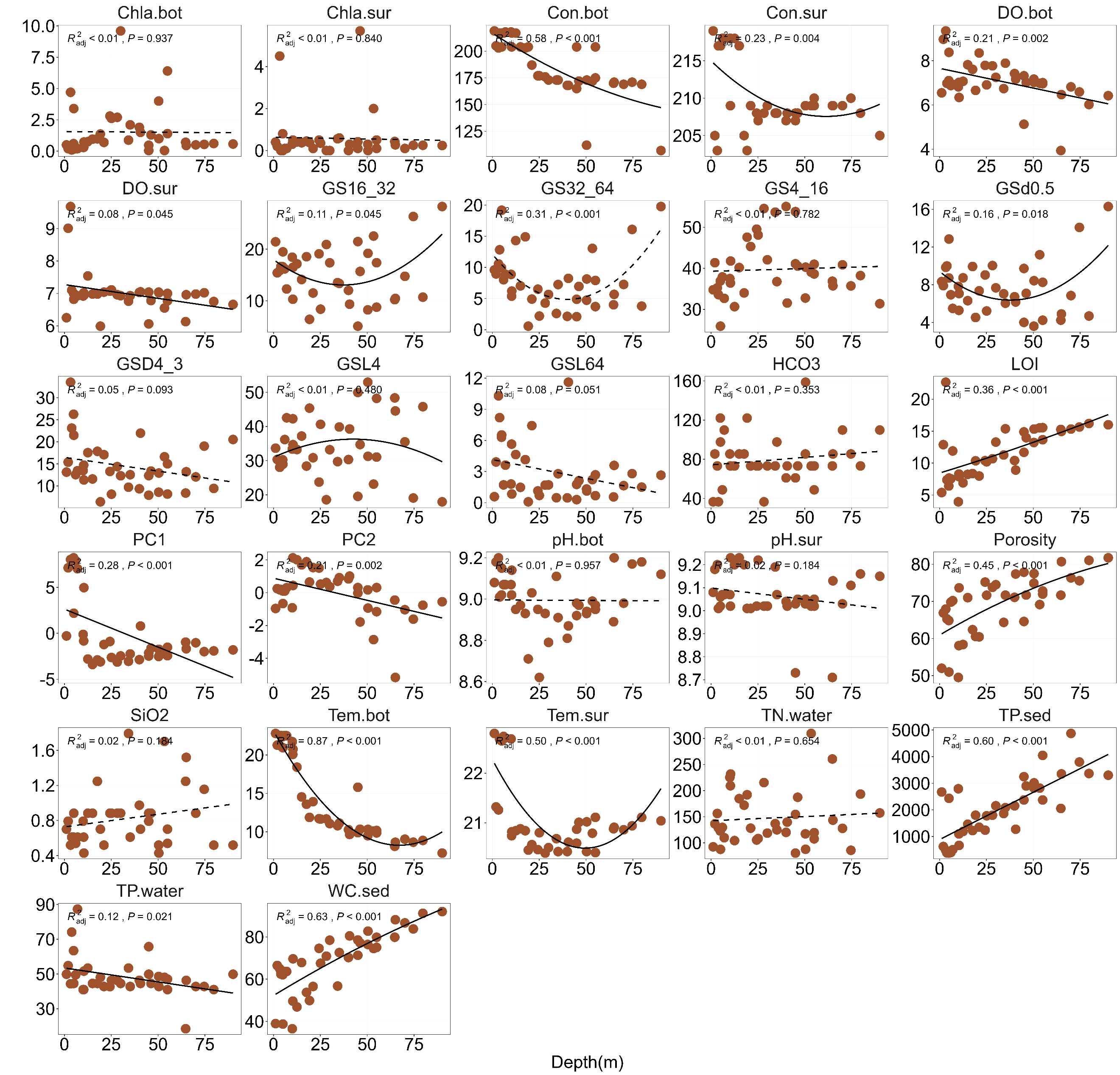


**Figure S4**. Water-depth diversity patterns for other environmental factors. We considered the relationships between other environmental factors and water depth. The relationships were modelled using linear and quadratic models. The better model was selected based on the lower value of Akaike's information criterion.


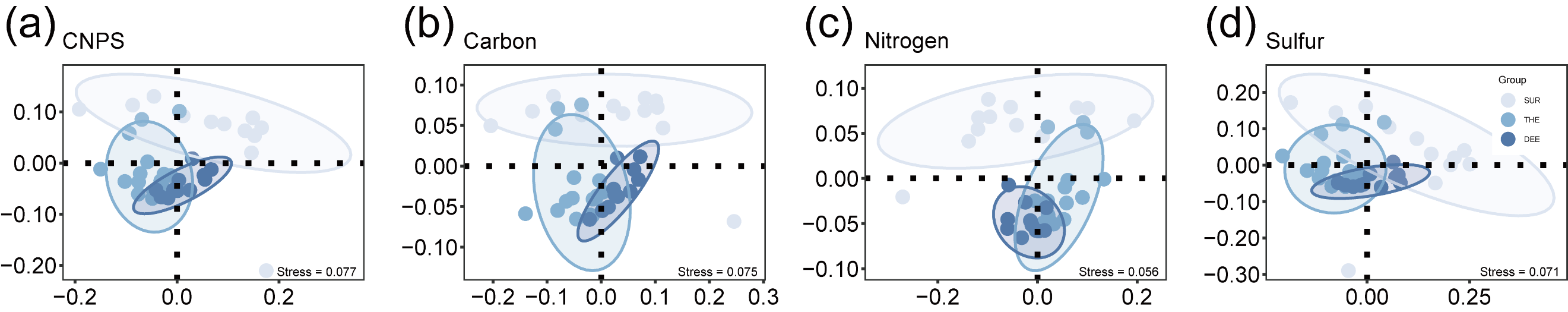


**Figure S5**. **Nonmetric multidimensional scaling (NMDS) plots of total functional genes (a) and three categories of functional genes (b-d).** Each point on the graph represented a sample, which was coloured according to water depth, scaling from light blue to dark blue.


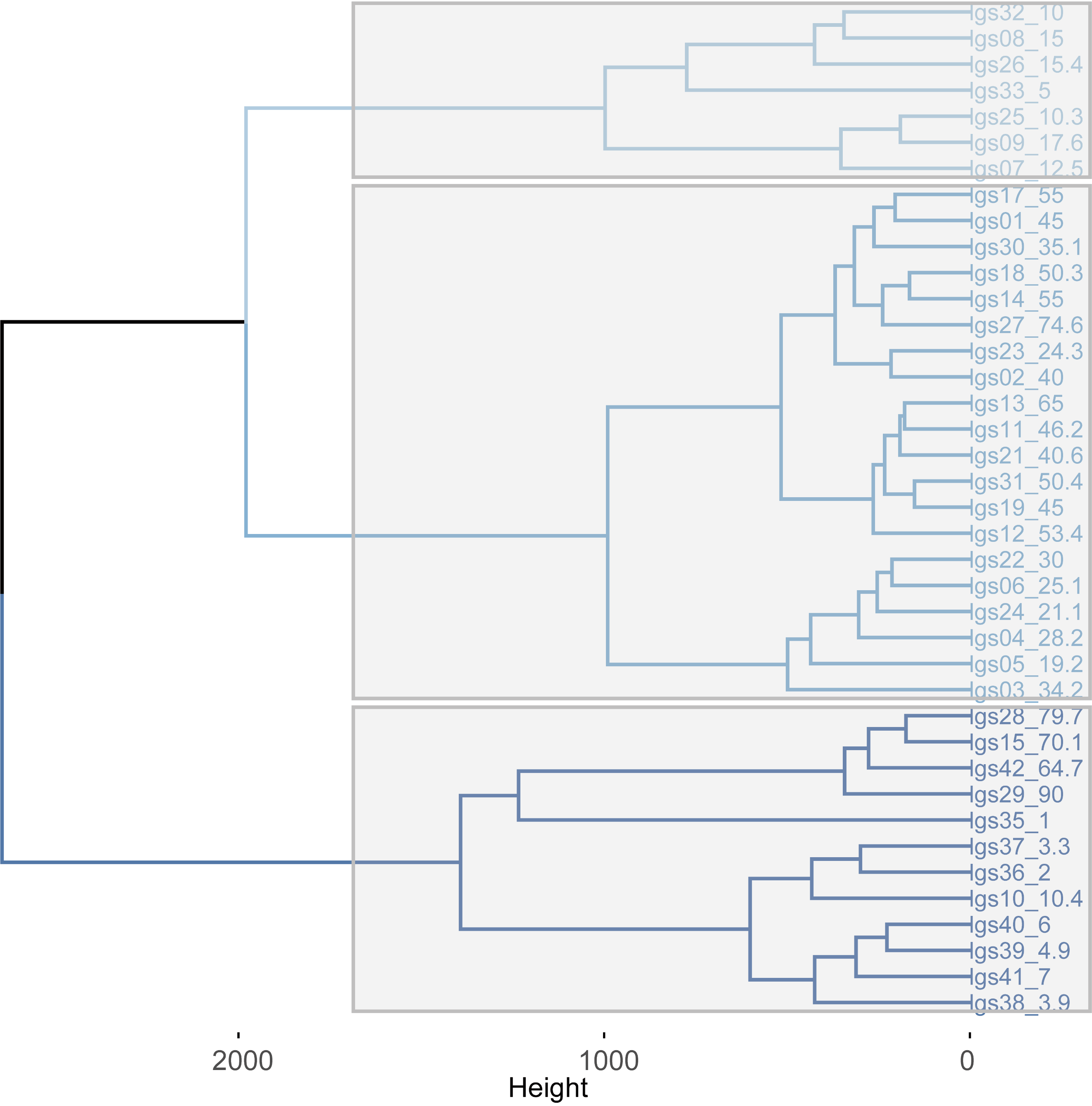


**Figure S6. Hierarchical clustering analysis of samples along the water depth gradient.** The dendrogram illustrated the clustering of samples based on functional gene composition across different depths. Samples are coloured from light blue to dark blue according to their respective layers: surface (SUR), thermocline (THE), and deep (DEE). The clustering was performed using Euclidean dissimilarity and the ward.D2 method. Branch lengths represent the degree of dissimilarity between samples, with shorter branches indicating greater similarity.


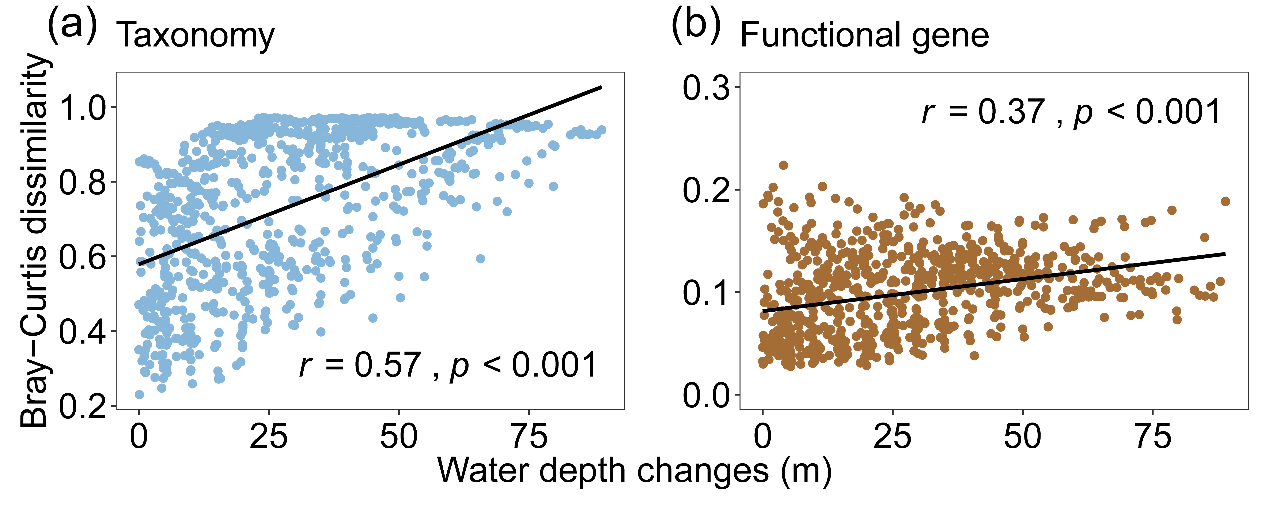


**Figure S7**. **Relationships between differences in functional gene composition and water depth changes.** The panels show the relationships between the Bray-Curtis differences of taxonomy (a) and functional gene (b) with water depth changes. The regression of the linear relationship based on the Gaussian generalized linear model is shown with a solid line. Mantel tests were used to examine correlations between differences in functional gene composition and differences in community composition using 9,999 permutations. The p and r values of the Mantel statistics are shown.


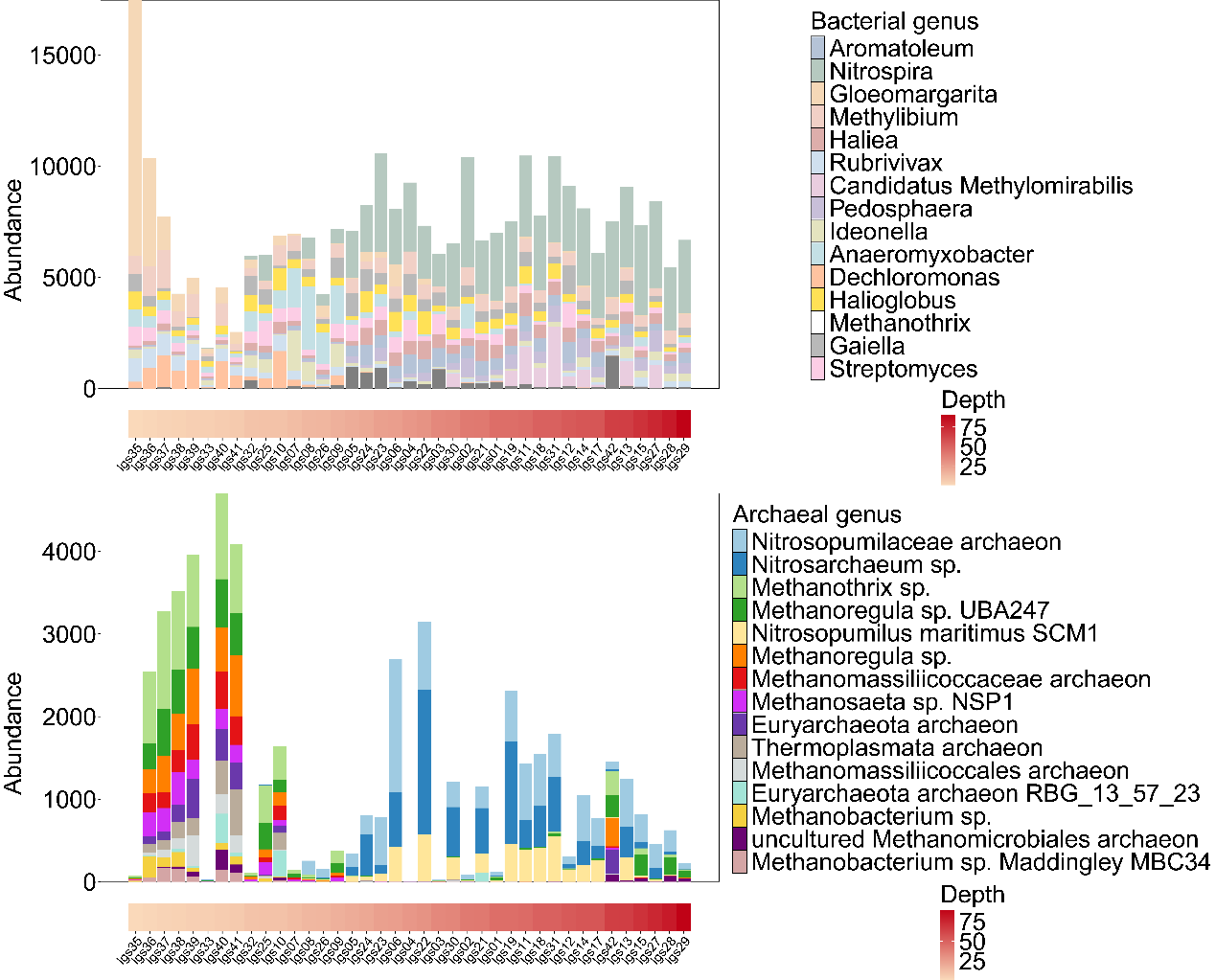


**Figure S8**. **The composition of bacterial and archaeal groups across different water depth.** The relative abundance of (a) bacterial genus and (b) archaeal genus in samples from different water depth. Only representative top 15 microbial genus with high relative abundances were annotated in the figure.


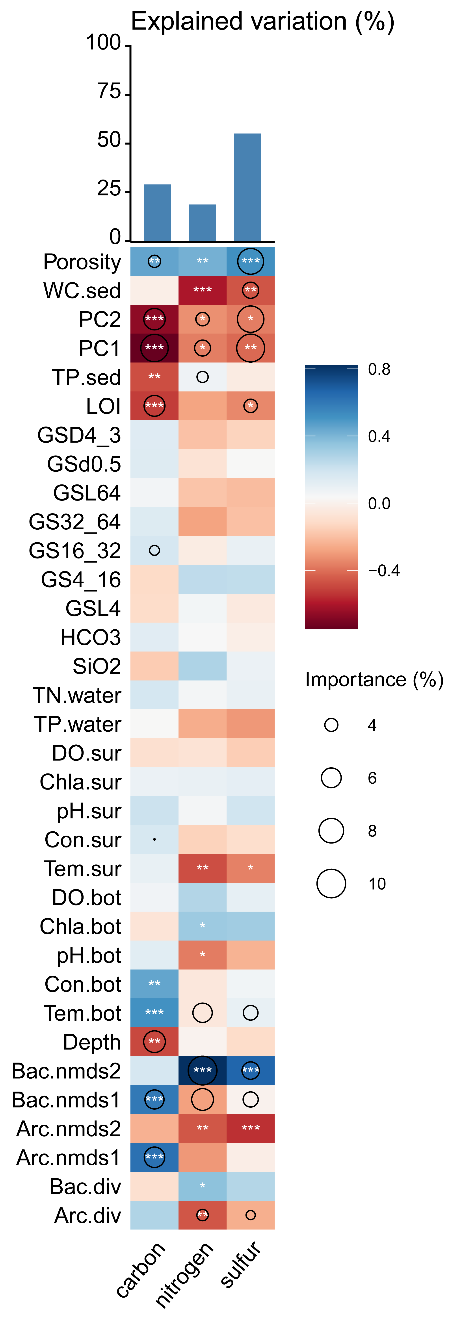


**Figure S9**. **Environmental factors driving the functional gene abundance.**  The impact of environmental factors on the abundance of functional gene subgroups. The strength of Spearman correlation is shown with the change in colour, and the significance is indicated by Asterisks. The colour gradient on the right represents the spearman rank correlation coefficient, with higher positive values (dark blue) indicating stronger positive correlation, and higher negative values (dark red) indicating stronger negative correlation. ****P* < 0.001, ***P* < 0.01, **P* < 0.05. The importance of environmental factors in explaining functional gene through random forest analysis was represented by circles of different sizes.

**
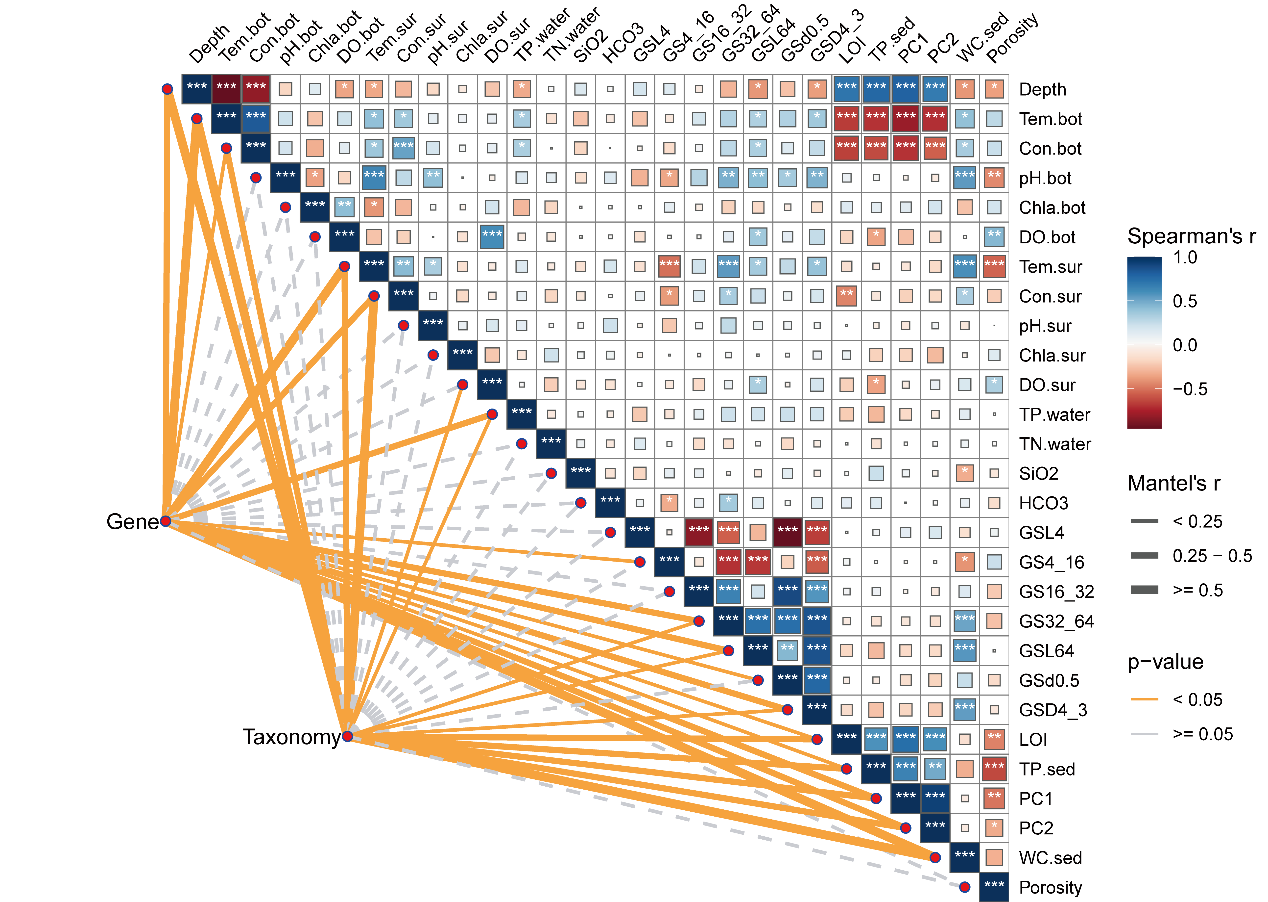
 Figure S10**. **The relationships between environmental factors and taxonomy and functional gene.** They were associated with each environmental factor by Mantel test. Edge width corresponds to the Mantel statistic r of the corresponding distance correlation, and edge colour indicates statistical significance. Blue and red show positive and negative relationships between two variables, respectively. The colour is darker, the relationships are stronger. The colour is proportional to the spearman correlation coefficient (dark blue, *r* = 1, dark red, *r* = -1). The details of abbreviations of environmental factors are available in Table S3.


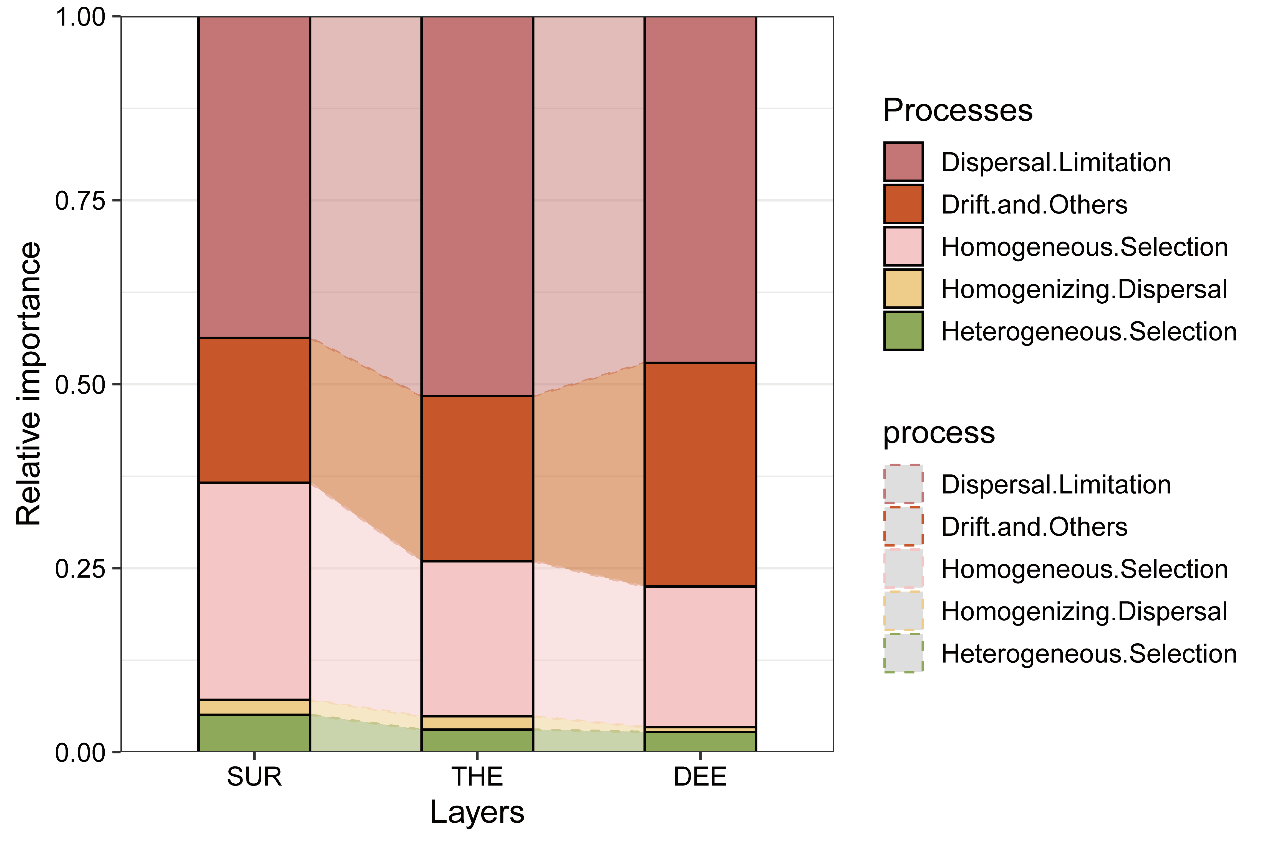


**Figure S11**. **Relative importance of ecological processes to microbial community assembly across different layers.** The stacked bar chart illustrated the proportional contributions of ecological processes, including dispersal limitation, drift, homogeneous selection, homogenizing dispersal, and heterogeneous selection to microbial community assembly in three depth layers: surface layer (SUR), thermocline (THE), and deep layer (DEE). Each bar represented a specific depth layer, with colours corresponding to different ecological processes. The relative importance was expressed as percentages of the total.


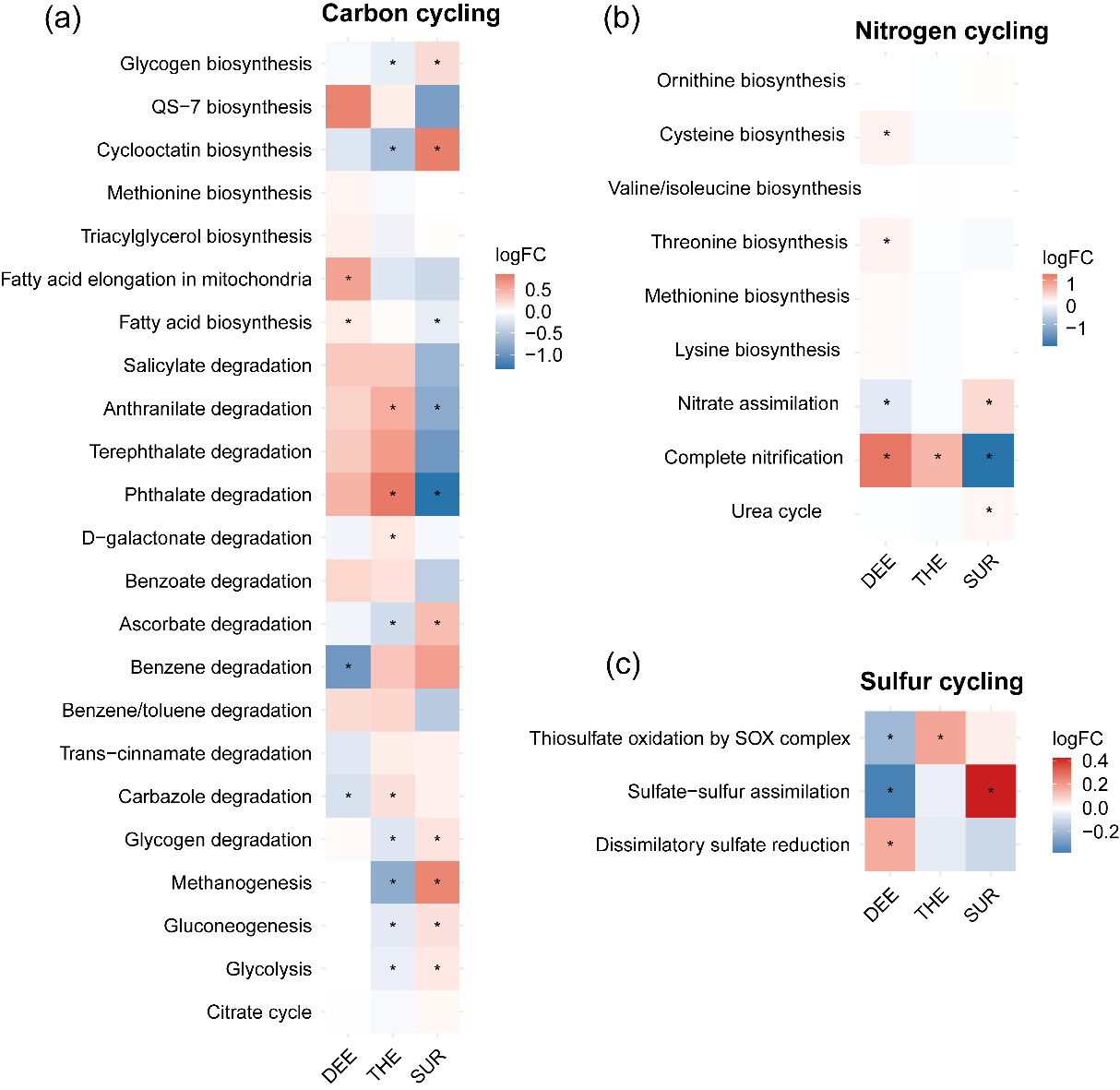


**Figure S12**.**Enrichment of functional pathways involved in C, N and S cycling among three layers.** Heatmap showed the enrichment of functional pathways involved in (a) carbon cycling, (b) nitrogen cycling, and (c) sulfur cycling among three water layers. Statistical significance of the changes in abundance was assessed by generalized linear model with a negative binomial distribution using edgeR100 package. P values were obtained from two-sided Likelihood Ratio Tests (LRTs) and adjusted for multiple comparisons via the Benjamini-Hochberg false discovery rate (FDR) procedure. Pathways with significant changes in abundance (*P* < 0.05) are indicated with an asterisk. LogFC: log2-fold change. SUR, surface layer; THE, thermocline layer; DEE, deep layer.
